# Detection of Transition Zone in Bowel Obstruction via Curved Multiplanar Reformations with Multidetector Computed Tomography

**DOI:** 10.7759/cureus.4233

**Published:** 2019-03-11

**Authors:** Maria Hassan, Muhammad Ali, Muhammad K Shazlee, Sanobar Bughio, Farheen Raza, Fahd Haroon

**Affiliations:** 1 Radiology, Dr. Ziauddin Hospital, Karachi, PAK; 2 Radiology, The Indus Hospital, Karachi, PAK

**Keywords:** mdct, curved mpr, multiplanar reformations, zone of transition, intestinal obstruction

## Abstract

Objective

We conducted this study to determine the added value of curved multiplanar reformations (CMPR) and multiplanar reformations (MPR) of multidetector computed tomography (MDCT) scan in the visualization and localization of the zone of transition in patients with intestinal obstruction.

Materials and methods

A total of 100 patients with suspected bowel obstruction were evaluated in a retrospective cross-sectional study from September 2016 to September 2018 at Dr. Ziauddin University Hospital, Clifton Campus. All patients underwent multidetector computed tomography (CT) scans with oral and intravenous contrast before surgical exploration. CMPR and MPR were acquired at the time of examination in each patient in addition to routine axial images. The CT scans were analyzed by two independent, experienced radiologists skilled at detecting the zones of transition in patients with bowel obstruction using the axial images alone, followed by axial images along with MPR, and then MPR plus CMPR. Patient data were masked to the radiologists. The CT scan findings were compared with surgical findings to determine the accuracy of CMPR in detecting the zone of transition between distended and collapsed bowel loops. The added CMPR showed high accuracy in the diagnosis of intestinal obstruction with a remarkable advantage over the conventional axial images. Data analysis was done on IBM SPSS Statistics for Windows, Version 20.0 (IBM Corp., Armonk, NY). Cohen’s kappa statistics were obtained to show the measure of agreement between the two readers. McNemar’s test was also applied to determine the homogeneity.

Results

Two radiologists, one with two years of experience and the other with five years of experience were 80% and 81% accurate, respectively, in identifying the zones of transition using axial images alone. Using axial images plus MPR, their accuracy was 88% and 92%, respectively. Using MPR plus CMPR, their accuracy was 96% and 98%, respectively. The accuracy of MPR plus CMPR views was significantly increased when compared to the accuracy using axial images alone. CT findings were compared to surgical findings in terms of diagnostic performance. The kappa value of 0.6 indicates moderate association and substantial agreement between two radiologists. McNemar’s test showed homogeneity in the number of valid cases.

Conclusion

CMPR is an important and accurate technique for evaluating intestinal obstruction in addition to MPR as it helps in better localization of the zone of transition and in determining the cause of obstruction. This insight provides guidance for the appropriate treatment.

## Introduction

Intestinal obstruction is a major surgical emergency [1], accounting for 20% of surgical admissions and is a major cause of morbidity around the world [2]. Intestinal obstruction is a relatively common condition, with its diagnoses based on clinical signs and symptoms, patient history, and radiographic findings. Conventional radiography is the first imaging modality used in patients of intestinal obstruction. However, the accuracy of this modality in determining the presence of an obstruction is still 46% to 80% [3-6]. Once the intestinal obstruction is diagnosed, the site and cause of obstruction with the presence of ischemia must be determined for appropriate treatment.

The development of fast imaging modalities like helical computed tomography (CT) allows for a more accurate assessment of abdominal organs [7]. Beyond helical CT, multidetector computed tomography (MDCT) is superior in that it shows both the extent of the disease and provides valuable insight for surgical planning based on the coronal and sagittal reformatted images [8]. Multiplanar reformation (MPR) is a new technology that helps in identifying the site, level, and cause of obstruction when classic axial findings are indeterminate [9-11]. Localizing the zone of transition is important in diagnosing patients with compromised arterial flow to the bowel which causes ischemia, necrosis, and perforation. Knowing the location of the transition zone helps guide operative planning and informs the choice between laparotomy and laparoscopic surgery. It is challenging to localize the zone of transition in patients with intestinal obstruction using axial slices alone. Furthermore, finding the zone of transition is also challenging in thin, lean patients in whom the interfaces between bowel loops are very thin due to the paucity of intraperitoneal fat [12-13].

We conducted this study to establish the added value of curved multiplanar reformations (CMPR) in the visualization of the zone of transition in intestinal obstruction patients using surgical findings as the gold standard. Our data was analyzed retrospectively, and we explored the future utility of CMPR in the evaluation of intestinal obstruction. A previous, smaller version of this study using fewer patients was presented at the 2017 Conference of Radiological Society of Pakistan (Abstract: Ali M, Shazlee MK, Raza F, Bughio S, Lal S: P-134 MDCT Diagnosis of Intestinal Obstruction: Added Value of Curved Multiplanar Reformats in Detecting Zone of Transition. 33^rd^ Annual Conference of Radiological Society of Pakistan; October 27 to 29, 2017).

## Materials and methods

A total of 140 patients with suspected bowel obstruction were evaluated in a retrospective cross-sectional study from September 2016 to September 2018 at Dr. Ziauddin University Hospital, Clifton Campus. All patients under clinical suspicion of bowel obstruction underwent MDCT scan with oral and intravenous (I/V) contrast before surgical exploration. One hundred patients (53 male patients, 47 female patients) were confirmed to have bowel obstruction after MDCT examination, and all of them underwent subsequent surgical exploration in our hospital. The age range of the patients was 6 to 82 years (mean age, 50 years ± 20 years). We used nonprobability purposive sampling techniques.

Informed consent was waived as the study is retrospective in nature. This study included patients presenting with symptoms of bowel obstruction including abdominal pain, abdominal distention, vomiting or constipation. Patients lost to follow-up, and those conservatively managed were excluded from this study.

The abdominal CT scans spanned the dome of the diaphragm to the symphysis pubis and were performed using a multidetector ASTEION™ 16 (Toshiba, Japan) with 5-mm slice thickness. All patients underwent MDCT scan with oral and I/V contrast, portovenous phase before surgical exploration with protocol collimation of 2 mm, pitch of 1.35, at 120 kvp, 250 mAs, and medium field of view. MPR with CMPR were acquired in each patient along with routine axial images. After establishing the diagnosis of obstruction, we detected the cause of obstruction with the zone of transition through MRP and CMPR techniques. The CT scan findings were compared with the surgical findings to determine the accuracy of CMPR in detecting the zone of transition between distended and collapsed bowel loops.

Images with masked patient data were reviewed by two radiologists (one with two years of experience and the other with five years of experience). First, the radiologists reviewed axial slices alone to determine the presence of bowel obstruction. They next reviewed axial slices plus MPR to determine bowel obstruction. Finally, they reviewed MPR plus CMPR to locate the zone of transition. All patients were monitored via follow-up evaluations. The surgical findings of all patients were collected and compared with the CT findings.

Data were registered on proforma, then transferred to IBM SPSS Statistics for Windows, Version 20.0 (IBM Corp., Armonk, NY) for statistical analysis. We evaluated the site, level, and severity of bowel obstruction. Later, using the 2 × 2 cross tabulation accuracy of axial alone, the axial plus MPR and MPR plus CMPR were calculated using surgical findings as the gold standard. We determined the kappa value to assess the agreement between the two radiologists. We used McNemar’s test to determine the homogeneity between the different data sets. A p-value <0.05 was considered statistically significant.

## Results

The most common presenting concern was abdominal distention (30%) followed by vomiting (23%), abdominal pain (22%), and constipation (12%). The most common cause of intestinal obstruction was adhesions (35%), followed by mesenteric ischemia (14%), hernia (13%), carcinoma (7%) and tuberculosis (7%) (Table 1).

**Table 1:** Cause of bowel obstructions

Cause of Obstruction	Frequency (%)	Percent (%)	Valid Percent (%)	Cumulative Percent (%)
Abdominal collection	2	2.0	2.0	2.0
Adhesion	35	35.0	35.0	37.0
Appendicitis	1	1.0	1.0	38.0
Carcinoma	7	7.0	7.0	45.0
Diverticulitis	1	1.0	1.0	46.0
Foreign body	3	3.0	3.0	49.0
Hernia	13	13.0	13.0	62.0
Ileoileal intussusception	1	1.0	1.0	63.0
Inflammatory	4	4.0	4.0	67.0
Mesenteric Ischemia	14	14.0	14.0	81.0
Perforation	3	3.0	3.0	84.0
Polyp	2	2.0	2.0	86.0
Pregnancy	1	1.0	1.0	87.0
Tuberculosis	7	7.0	7.0	94.0
Volvulus	6	6.0	6.0	100.0
Total	100	100.0	100.0	

The radiologist with two years of experience and the radiologist with five years of experience were 80% and 81% accurate, respectively, in identifying the zones of transition using axial images alone. Using axial images plus MPR, their accuracy was 88% and 92%, respectively. Using MPR plus CMPR, their accuracy was 96% and 98%, respectively (Table 2).

**Table 2:** Accuracy in locating the zone of transition with various post-processing techniques P<0.05 is statistically significant. MPR, multiplanar reformation; CMPR, curved multiplanar reformation.

	Axial Images	Axial plus MPR	MPR plus CMPR	Improvement by CMPR (with respect to axial images alone)
Radiologist 1	80/100(81%)	88/100(88%)	96/100(96%)	15%
Radiologist 2	81/100(80%)	92/100(92%)	98/100(98%)	18%
McNemar Values	1.000	0.219	0.500	
Measure of Agreement(Kappa)	0.635	0.668	0.658	

The accuracy of using MPR with CMPR views was significantly increased when compared to the accuracy of using axial images alone. CT findings were compared to surgical findings in terms of diagnostic performance.

Cohen’s kappa was applied to measure the association between the two radiologists. The kappa value was 0.6, indicating a moderate association and a substantial measure of agreement between the two radiologists. McNemar’s test indicated a homogeneity in the number of valid cases using binomial distribution, as seen in Table 2.

## Discussion

CT technology has shown major advances since its initial development, and MDCT is the latest outcome. MDCT is capable of reforming two- and three-dimensional images with high quality in the z-axis. This improves the quality of images, significantly reduces the examination time, and allows for well-defined perfusion phases in depicting organs.

Several studies have proven the value of CT in confirming diagnoses (at both site and spinal level) and revealing the cause of small intestinal obstruction. These studies report a sensitivity of 94% to 100% and an accuracy of 90% to 95%. CT scanning indicates the site, level, and severity of obstruction and is sensitive for the signs of strangulation and volvulus [10-11]. Bowel obstruction is a dynamic process-it can rapidly develop into a catastrophe, or it can resolve by itself. Fully understanding the severity of ischemia, the cause of obstruction, and the site of transition aids the surgeon in operative planning [12-13].

There are multiple causes of intestinal obstruction such as adhesions, mesenteric ischemia, hernias, and carcinomas. Causes also include inflammatory conditions such as appendicitis and sigmoid diverticulitis. Intussusception is a common cause for intestinal obstruction in children, but it is uncommon in adults. Intraluminal causes of obstruction include foreign bodies like gossypiboma, fetus, gall stones, and bezoars [14].

MPR images played a limited role in previous studies, and only a few studies have suggested MPR’s significance. Jaffe et al. proved that coronal reformation significantly increased both interobserver agreement and confidence levels for diagnosing intestinal obstruction, but they did not comment on the value of MPR in locating the zone of transition [15]. Hodel et al. reported an accuracy of 93% by using MPR to locate the transition zone [16]. A study by Keoplung et al. showed that MPR helped increase diagnostic confidence and confirmed findings found on axial images [17].

Wang showed that with the two readers, the accuracy of transverse images alone in locating small bowel obstruction was 79% and 78%, the accuracy using transverse plus coronal images was 90% and 92%, and the accuracy using MPR plus CPR views was 97% and 99%, respectively-values quite similar to the results of this study (Abstract: Wang Y. Detection of Transition Zone in Patients with Small Bowel Obstruction: Added Value of Curved Planar Reformations from Isotropic Voxels with 64-MDCT. European Society of Radiology [ESR. ECR 2012 book of abstracts-b-scientific sessions. Insights into imaging]. 2012, 3:135. 10.1594/ecr2012/C-0905).

MPR is an excellent technique as it allows for viewing in multiple planes. CMPR is similar to MPR, and it allows for the creation of a single-voxel that enables visualization of a curved structure by marking points manually along the structure of interest. CMPR is useful for displaying tubular structures. However, a disadvantage of CMPR is its high dependence on accurate selection of the curve.

The additional curved reformatted images correctly identify single or multiple zones of transition between dilated and collapsed segments of bowel. First, the transition point needs to be viewed from a variety of perspectives which increases the radiologist’s diagnostic confidence level. The next step is differentiating between simple obstruction or complicated obstruction, such as a closed-loop obstruction that causes ischemia or incarceration to the bowel requiring urgent action for the patient’s health. Signs of ischemia include congestive changes in the mesentery associated with the affected bowel and have a misty appearance. Intestinal ischemia and infarction are the major causes of morbidity and mortality in patients with bowel obstruction [18]. In patients with strangulation, surgery performed within 36 hours of symptom onset has a reported mortality of 8%, whereas surgical delays beyond 36 hours increase the mortality up to 25% [18]. Therefore, timely diagnosis of bowel obstruction is critical.

## Conclusions

CMPR is an important and accurate technique for evaluating intestinal obstruction in addition to MPR as it helps in better localization of the zone of transition and in determining the cause of obstruction. Therefore, the insight gained from adding CMPR to MPR provides guidance for the appropriate treatment to achieve potentially better outcomes for patients.
